# Evaluation of the antimicrobial and cytotoxic activity of nerolidol encapsulated in a nanoliposome system

**DOI:** 10.3389/fvets.2025.1641746

**Published:** 2025-10-27

**Authors:** Nicolò Mezzasalma, Costanza Spadini, Federico Righi, Marica Simoni, Gaetano Lamberti, Anna Angela Barba, Dante Greco, Alessia Merelli, Lorenzo Bosio, Alessandro Cupola, Emiliana Schiano, Simone Taddei, Clotilde Silvia Cabassi

**Affiliations:** ^1^Department of Veterinary Science, University of Parma, Parma, Italy; ^2^Department of Industrial Engineering, University of Salerno, Fisciano, Italy; ^3^Enhanced Systems and Technology Srl, Avellino, Italy; ^4^Department of Pharmacy, University of Salerno, Fisciano, Italy; ^5^Farmer Spa R&D Department, Mantova, Italy

**Keywords:** alternative antimicrobials, liposome, time-kill assay, livestock, plant feed additives, animal nutrition

## Abstract

Plant-derived compounds have emerged as potential alternatives to traditional antimicrobials in livestock; however, their application may be limited by degradation in the gastrointestinal tract. Nanoliposome encapsulation offers a strategy to overcome these limitations. In this study, we investigated the effects of nerolidol encapsulation, by evaluating the antimicrobial activity of free-nerolidol (NER), nerolidol-loaded nanoliposomes (LN), and unloaded nanoliposomes (UN) (Lipobox™) using a Time-Kill assay. The cytotoxicity of these formulations was assessed through MTT assay on swine and bovine cell lines. NER was effective against MRSA, *Enterococcus faecium*, and *Lactobacillus acidophilus* at all time points, at concentrations ≥62.5, ≥15.63 and ≥1,000 μg/ml, respectively, but was ineffective against Gram-negative bacteria Conversely, LN and UN were effective against all bacteria, showing the best activity at 2,500 μg/ml. LN showed the greatest activity against MRSA up to 6 h while UN on *E. faecium* up to 4 h (*P* < 0.05). No difference between LN and UN on *Salmonella Typhimurium* up to 24 h and on *E. coli* up to 6 h at this concentration (*P* > 0.05) was observed. For *L. acidophilus*, both LN and UN were effective up to 6 h even at the lowest concentration (9.77 μg/ml). NER showed high cytotoxicity on MDBK and IPEC-J2 cells at all doses; while LN and UN were low-toxic at concentrations ≤ 1,250 μg/ml or ≤ 625 μg/ml, respectively. These results suggest that nanoliposomes themselves exhibit dose-dependent antimicrobial and cytotoxicity activity; however, when NER is encapsulated its spectrum of activity its enhanced.

## 1 Introduction

Prior to their ban as growth promoters (GPs) in animal feed within the EU in January 2006 (1), antibiotics were extensively employed in livestock production for both therapeutic purposes and to enhance animal growth and productivity (2). However, such practices significantly contributed to the emergence and spread of antimicrobial resistance (3). The progressive phasing out of antibiotics for non-therapeutic applications aligns with the growing emphasis on sustainability in animal production systems and has stimulated the search for natural alternatives. Among these, nutraceutical compounds—comprising microbial and plant-derived products—have garnered increasing interest from both the scientific community and the animal feed industry (4).

The use of plant-derived substances as feed additives, commonly referred to plant-derived feed additives (PFAs), represents a well-established approach in animal nutrition (5). The most widely utilized PFAs include essential oils, plant extracts, and their associated bioactive compounds. The appeal of PFAs is in their broad spectrum of biological activities, such as antioxidant (vitamin-like), metabolic (e.g., hepatoprotective), and antimicrobial effects, which make them valuable candidates for veterinary applications (6–8). Despite the promising antimicrobial potential of various essential oils, their practical application in animal diets may be constrained by the high volatility, chemical instability, and susceptibility to auto-oxidation of their principal active constituents (9). Furthermore, the composition and efficacy of essential oils and plant extracts can be highly variable, influenced by intrinsic factors (e.g., plant part used, harvest season, geographic origin) and extrinsic factors (e.g., extraction method, drying, and storage conditions) (10). An emerging alternative to essential oils is represented by nature-identical compounds (NICs), which are synthetic analogs of essential oils bioactives. NICs offer advantages such as enhanced chemical stability, consistent antimicrobial efficacy over time, and batch-to-batch uniformity, positioning them as a promising substitute for conventional antibiotics in animal nutrition (6).

Sesquiterpenes, known for their diverse biological activities, represent a particularly promising class of NICs (11). Among them, nerolidol (NER) has garnered significant attention. The NER is a naturally occurring sesquiterpene alcohol found in the essential oils of various arboreal and shrubby plant species, such as *Piper claussenianum* (Miq.) C. DC., *Momordica charantia* L., *Ginkgo biloba* L., *Baccharis dracunculifolia* DC., and *Myrocarpus frondosus*, as well as in several flowering plants including lavender, neroli, lemongrass, tea tree, and ginger (12, 13). This compound exhibits a broad spectrum of bioactivities, with applications in the pharmaceutical, food, and cosmetic industries due to its antimicrobial, anti-inflammatory, and antibiofilm properties (14). Despite its potential, the application of NER in animal nutrition faces several challenges. These include its low aqueous solubility—limiting systemic bioavailability—cytotoxic potential, and rapid absorption and degradation in the gastrointestinal tract of livestock (11, 15).

To overcome these limitations and improve the delivery and efficacy of NICs such as NER, encapsulation technologies have been explored (15, 16). Encapsulation can protect active compounds from premature degradation in the gastrointestinal environment and enhance their bioavailability. Various encapsulating materials have been investigated, including natural proteins (e.g., albumin, gelatin), polysaccharide-based polymers (e.g., arginase, hyaluronic acid, chitosan), and lipid-based systems such as liposomes. The effectiveness of encapsulation depends largely on the choice of matrix material and the specific preparation methods employed (15, 17). Liposomes are among the most widely adopted encapsulation systems for essential oils. Composed of phospholipid bilayers, they form spherical vesicles with diameters typically ranging from 25 to 1000 nm; vesicles between 50 and 150 nm are referred to as nanoliposomes (18). Liposomes offer several advantages, including high encapsulation efficiency, simple preparation under mild conditions, reproducibility, and controlled release of encapsulated bioactives (19). Essential oils encapsulated in liposomes have demonstrated enhanced stability and biological activity. For instance, *Origanum* essential oil encapsulated in liposomes exhibited improved antimicrobial efficacy against several human pathogens, including *Staphylococcus aureus, Pseudomonas aeruginosa, Escherichia coli, Enterobacter cloacae*, and *Klebsiella pneumoniae*. Similarly, essential oils from *Artemisia arborescens* showed antiviral activity against herpes simplex virus type 1 when delivered via liposomal systems (16). NER has been encapsulated—either alone or in combination with cyclodextrins—using various delivery systems such as chitosan-alginate nanoparticles, cyclodextrins, and liposomes, particularly for applications in the food industry. These systems have yielded variable outcomes depending on the encapsulation method and formulation parameters NER was encapsulated, either alone or in combination with cyclodextrins, using chitosan-alginate nanoparticles, cyclodextrins, and liposomes (16, 17).

To the best of our knowledge, no studies have yet investigated the use of nanoliposomes loaded with nerolidol in the context of veterinary medicine. Considering this, the present study evaluates the time-dependent antimicrobial activity of NER-loaded nanoliposomes, in comparison to unloaded nanoliposomes and free NER, against main bacterial pathogens representative of the commensal microbiota in livestock. Concurrently, the cytotoxic effects of these formulations were assessed in both bovine and porcine cell lines. The aim was to determine if encapsulation of NER within nanoliposomes could enhance its antimicrobial efficacy in parallel with the reduction of its cytotoxicity with respect to the free form.

## 2 Materials and methods

### 2.1 Nerolidol nanoliposomes production through the simil-microfluidic apparatus

#### 2.1.1 Materials

L-a-Phosphatidylcholine (PC) from soybean (CAS no. 8002-43-5) was purchased from A.C.E.F. (powder soybean lecithin E322, Fiorenzuola D'Arda, PC); cholesterol (CHOL; CAS no. 57-88-5) was purchased from CRODA (Cholesterol USP-PW (RB)LD 02210/SAMP; Mortara, PV); ethanol of analytical grade (CAS no. 64-17-5) and Nerolidol (CAS no. 7212-44-4) were purchased from Sigma Aldrich (Milan, Italy).

#### 2.1.2 Manufacturing technique

Unloaded nanoliposomes (UN) and loaded nanoliposomes (LN) have been prepared using the simil-microfluidic technique developed and patented by the research group of the University of Salerno (20–23). Briefly, two feed solutions (lipids/ethanol/Nerolidol and water) were pushed by peristaltic pumps into the production section, a millimetric tubular device where the interdiffusion of the two flows leads to the formation of liposomes directly at nanometric scale. Specifically, the lipids/ethanol solutions were fed into a needle (0.6 mm internal diameter) inserted into the production section tube, a 3 mm internal diameter silicon tube, where water also was fed. The ethanolic solution was prepared using a ratio of 5:1 between PC and CHOL (2.35 g of PC and 0.47 g of CHOL in 50 ml of ethanol for UN). To obtain LN, 0.0285 g of NER were added to this solution. To obtain theoretical load ratio 1% (NER divided by the sum of lipids and NER itself). The production process was carried out using a ratio between the volumetric flow rates of 10:1 (i.e. 4.5 ml/min of ethanolic solution and 45 ml/min of water). By this way, a flowrate of roughly 3 L/h of nanoliposome suspension was produced. The concentration of lipids was roughly 5 g/L (*nominal* concentration of 5,000 μg/ml), and the NER concentration was roughly 0.05 g/L (load ratio of 1%, the tested encapsulation efficiency being close to 100%), giving a *nominal* concentration of 50 μg/ml.

### 2.2 Antimicrobial activity testing

#### 2.2.1 Bacterial strains and bacterial inoculum preparation

The antimicrobial evaluation of the tested compounds was performed against five reference bacterial strains of veterinary interest: Methicillin-Resistant *Staphylococcus aureus* (MRSA) ATCC 43300, *Escherichia coli* ATCC 25922, *S. enterica* subsp. *enterica* serovar Typhimurium ATCC 14028, *Enterococcus faecium* ATCC 19434 and *Lactobacillus acidophilus* ATCC 4356. All reference strains were purchased from ATCC^®^ (USA). The bacterial inoculum was prepared following the CLSI method (24). All microbiological assays were performed within 30 min after the inoculum's standardization. Five bacterial colonies from solid fresh cultures of each tested strain were inoculated into sterile tubes containing Müeller Hinton Broth (MHB) and incubated at 37 °C under aerobic conditions for 24 h, except for the *E. faecium* and *L. acidophilus*, which were incubated in microaerophilic conditions. After incubation, the bacterial suspension was centrifuged at 2,000 rpm at 4 °C for 20 min to separate the bacterial pellet from the supernatant. Then, the pellet was resuspended in 10 mM phosphate buffer (PB), pH 7. The bacterial suspension was adjusted in PB to obtain an optical density (OD) value in the range 0.08–0.13 at 600 nm in a 1 cm light path cuvette, approximately equivalent to a 10^8^ CFU/mL suspension. This suspension was further diluted 1:100 in sterile MHB. Fifty microliters of the bacterial suspension containing 10^6^ CFU/ml were inoculated into each well, to obtain a final concentration of 5 x 10^5^ CFU/ml. Bacterial suspensions were assessed through a Biophotometer plus (Eppendorf, Hamburg, Germany) spectrophotometer (k¼600 nm).

#### 2.2.2 Time-kill (TK) assay

The TK assay of NER, UN and LN was performed according to the literature (25, 26). The NER was initially dissolved in DMSO to prepare a stock solution at concentration of 400 mg/ml. Serial two-fold dilutions of the stock solution were prepared in DMSO. Then, 40 μl of each tested concentration was added to a tube containing 3.960 ml of MHB and a bacterial suspension of 5 × 105 CFU/ml, achieving final test concentrations ranging from 4,000 to 7.81 μg/ml.

Instead, for UN and LN, an initial stock suspension of 5,000 μg/ml, prepared as described above, was diluted two-fold in MHB medium. Subsequently, 2 ml of each diluted suspension was mixed with 2 ml of a bacterial suspension containing 10^6^ CFU/ml, resulting in a final range concentrations ranging from 2,500 to 9.77 μg/ml for both LN and UN, with a final bacterial concentration of 5 × 10^5^ CFU/ml.

Bacterial growth was quantified after 2, 4, 6 and 24 h of incubation at 37 °C in aerobic conditions for *Escherichia coli, S*. Typhimurium, and MRSA, and in microaerophilia for *Enterococcus faecium* and *Lactobacillus acidophilus*. Ten microliters were plated on Mueller–Hinton Agar (MHA) and the plates were incubated under aerobic/microaerophilic conditions at 37 °C for 24 h. After incubation, for each experimental point and tested concentrations, colonies were counted.

For each assay, three experiments, each comprised of three replicates, were performed including growth (GC) and sterility controls.

### 2.3 Cytotoxicity assay

The evaluation of cytotoxicity of NER, UN and LN were performed using MTT cell survival assay in according to the literature (27) on Madin-Darby bovine kidney (MDBK) ATCC CRL-6071 and Intestinal Porcine Enterocyte cells IPEC-J2 (BS CL 205 purchased by Biobanking - of Veterinary Resources of Istituto Zooprofilattico Sperimentale della Lombardia e dell'Emilia Romagna–Brescia-Italy). Briefly, regarding UN and LN, 50 μl of stock solution were added in a flat bottomed 96 wells sterile plate containing MDBK cells in Modified Eagle Medium (MEM) and Fetal Bovine Serum 10% (FBS) or IPEC-J2 cells in Dulbecco's Modified Eagle Medium, Hams's F-12 mixture (DMEM/F-12), and scalar dilutions were performed ranging from 2,500 to 9.77 μg/ml. Regarding NER, 1 μl of NER diluted on DMSO (4,000 to 31.25 μg/ml scalar dilutions) was added to each well.

Cell cultures were incubated for 4 h with 100 μl/well of MTT (5 mg/ml concentration) before the addition of 100 μl/ml of solubilization solution (10% SDS in HCL 0.01 M), and further incubated for 16 h at 37 °C. The OD was measured at 540 nm, using reading plates.

For each assay, three experiments, each comprised of three replicates, were performed including for each experiment, a negative control.

## 3 Statistical analysis

Statistical analyses were performed using IBM SPSS Statistics (Version 29.1; Armonk, NY: IBM Corp). To meet the assumptions of normality required for parametric testing, bacterial counts expressed as CFU/mL were log-transformed using a base-10 logarithmic transformation (log10).

The normality of the transformed data was confirmed using the Kolmogorov–Smirnov test.

Data regarding TK assay was analyzed using repeated-measures ANOVA, conducted through the General Linear Model (GLM) procedure. Mauchly's test indicated that the assumption of sphericity was violated (*P* < 0.05); therefore, degrees of freedom were corrected using the Greenhouse–Geisser estimate of sphericity.

*Post-hoc* comparisons were conducted using the Bonferroni test. A significant level of α = 0.05 was set for all statistical tests.

For the evaluation of cytotoxicity, differences with negative control were tested by the Student's *t* test.

## 4 Results

### 4.1 TK assay

**Figure 1:**
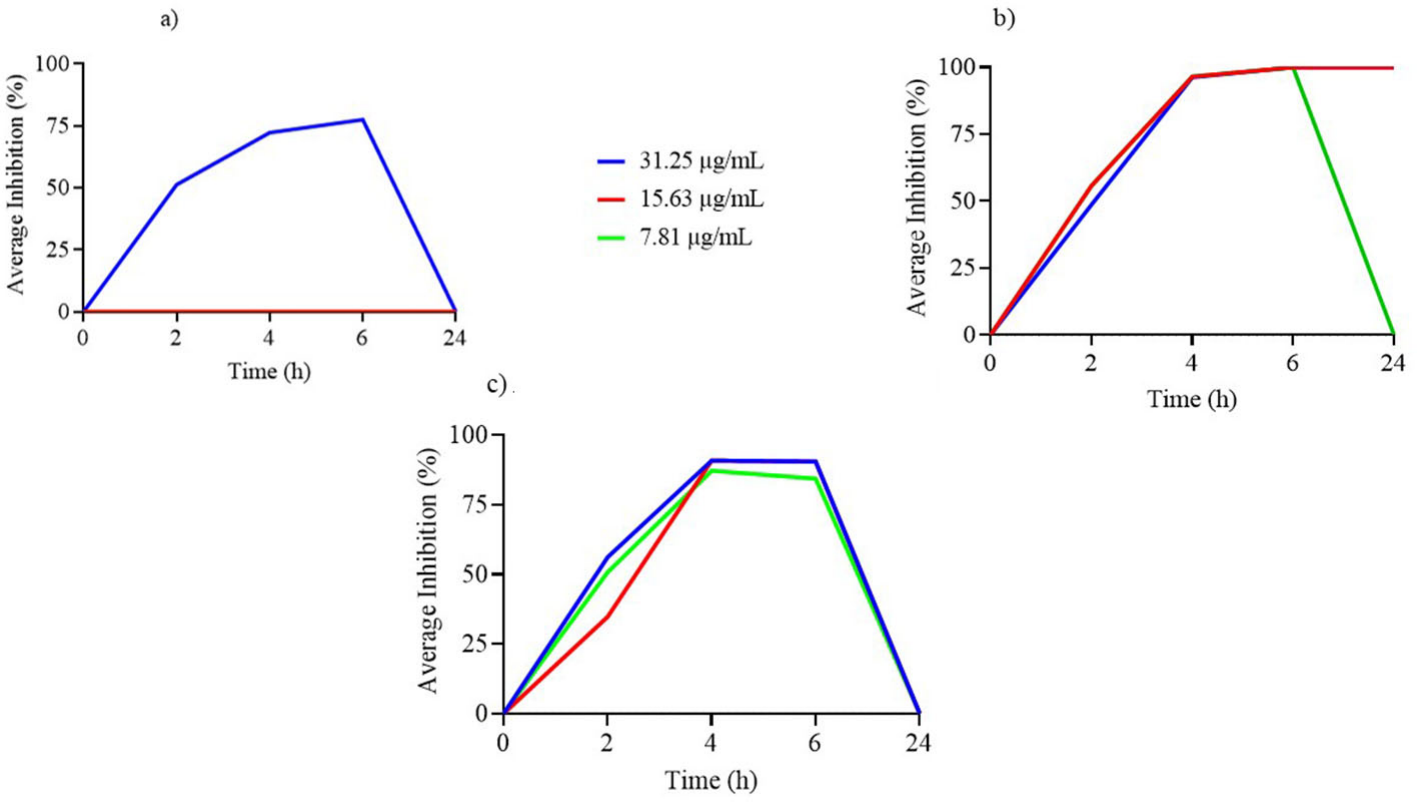
**(a–c)** Time-kill assay of nerolidol concentrations (μg/ml) incorporated in nanoliposome formulations against tested reference bacterial strains.

**Figure 2:**
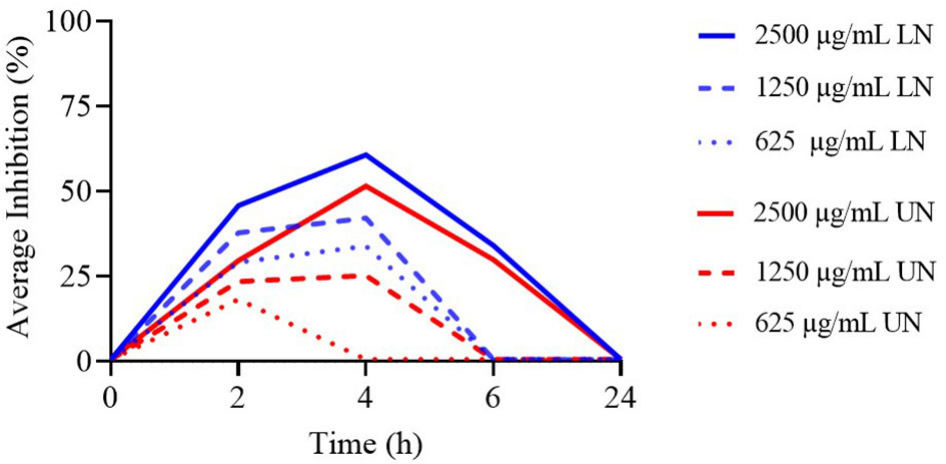
Time-Kill assay of different concentrations (μg/ml) of loaded nanoliposome (LN) and unloaded nanoliposome (UN) against Methicillin-Resistant *Staphylococcus aureus* ATCC 43300.

**Figure 3:**
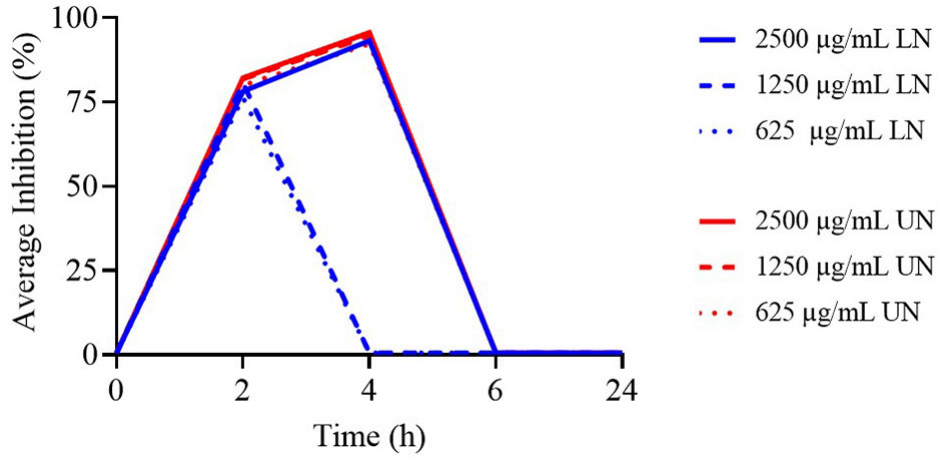
Time-Kill assay of different concentrations (μg/ml) of loaded nanoliposome (LN) and unloaded nanoliposome (UN) against *Enterococcus faecium* ATCC 19434.

**Figure 4:**
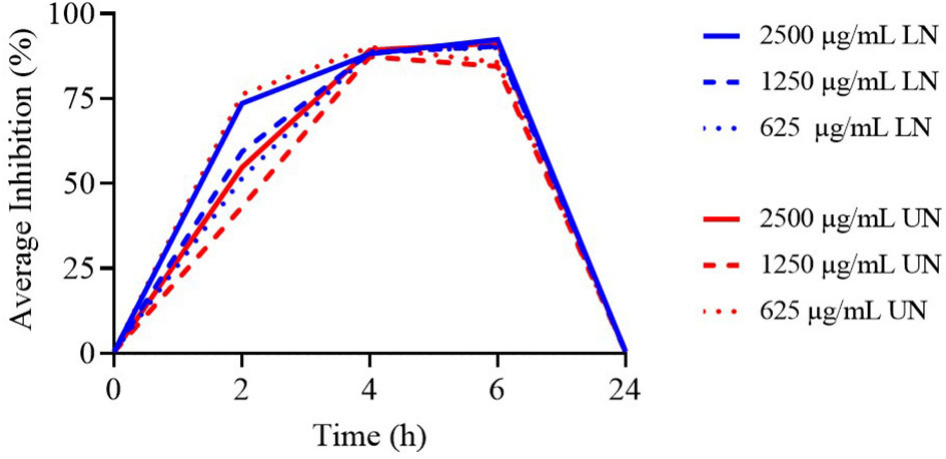
Time-Kill assay of different concentrations (μg/ml) of loaded nanoliposome (LN) and unloaded nanoliposome (UN) against *Lactobacillus acidophilus* ATCC 4356.

**Figure 5:**
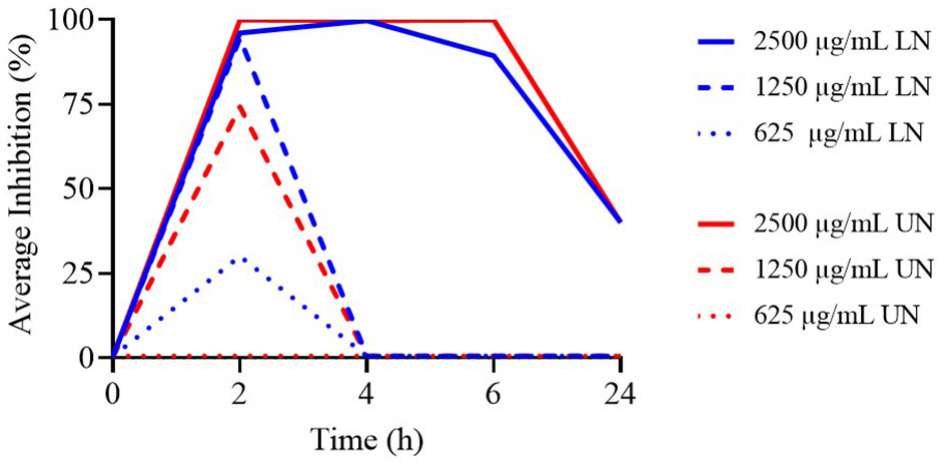
Time-Kill assay of different concentrations (μg/ml) of loaded nanoliposome (LN) and unloaded nanoliposome (UN) against *S*. Typhimurium ATCC 14028, at different time points.

**Figure 6:**
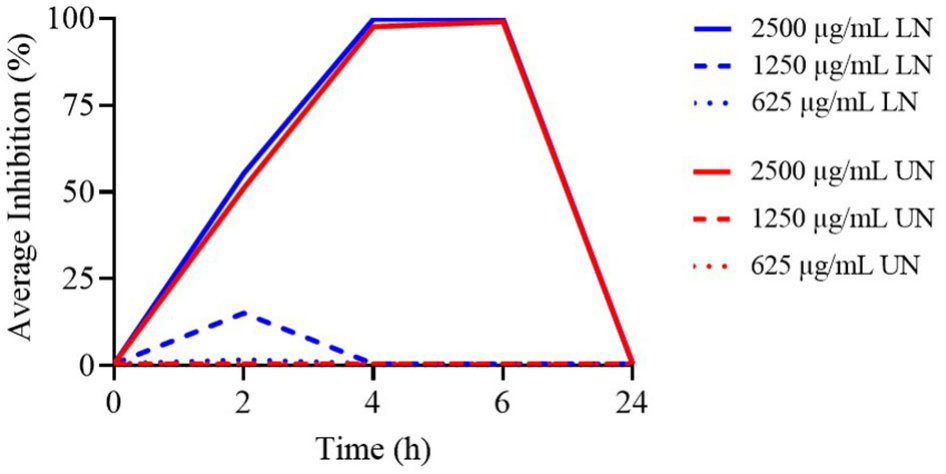
Time-Kill assay of different concentrations (μg/ml) of loaded nanoliposome (LN) and unloaded nanoliposome (UN) against *E. coli* ATCC 25922, at different time points.

The time-kill (TK) assay results for free nerolidol (NER) and its nanoliposome formulations at all the tested concentrations and experimental time points are provided in the Supplementary Materials (Supplementary Tables 1–8). Data for Gram-negative bacteria treated with free NER are not presented, as the treatments showed no measurable efficacy. The TK curves corresponding to selected concentrations directly comparable between free NER and the nanoliposome formulations are shown in Figures 1–6.

#### 4.1.1 Nerolidol

##### 4.1.1.1 Methicillin-resistant *Staphylococcus aureus* (MRSA) TK assay

The TK assay results for NER against methicillin-resistant *Staphylococcus aureus* (MRSA) across all tested concentrations are presented in Supplementary Table 1. NER concentrations ranging from 4,000 to 62.5 μg/ml resulted in a significant reduction in bacterial counts over time. In contrast, concentrations ≤ 31.25 μg/ml led to a significant increase in bacterial growth (*P* < 0.001). Statistically significant differences were observed at each time point among the tested concentrations (*P* < 0.001). At 2, 4, and 6 h, lower bacterial counts were recorded for concentrations of 250–62.5 μg/ml, 2,000–62.5 μg/ml, and 1,000–125 μg/ml, respectively, compared to 4,000 μg/ml. At 24 h, concentrations ≥125 μg/ml exhibited the highest antimicrobial activity (*P* < 0.001).

Figure 1a illustrates the TK curves for free NER concentrations equivalent to those used in the nanoliposome formulation. Notably, only the 31.25 μg/ml concentration demonstrated measurable antimicrobial activity, and this effect was limited to the first 6 h, with no activity observed at 24 h.

##### 4.1.1.2 Enterococcus faecium TK assay

The TK assay results for *Enterococcus faecium* are reported in Supplementary Table 2. All concentrations except 7.81 μg/ml significantly reduced bacterial counts over the experimental time course (*P* < 0.001). At 2 h, a significant reduction in bacterial count was observed for 62.5 μg/ml compared to concentrations ≥2,000 μg/ml (*P* < 0.001). At 4 h, concentrations of 125, 31.25, 15.63, and 7.81 μg/ml exhibited greater antimicrobial activity than 2,000 μg/ml (*P* < 0.001). At 6 h, the 62.5 and 31.25 μg/ml concentrations were more effective than both 7.81 μg/ml and ≥2,000 μg/ml (*P* < 0.001). At 24 h, concentrations ≥15.63 μg/ml maintained antimicrobial activity.

As shown in Figure 1b, the TK curves of free NER at concentrations included in the nanoliposome formulation indicate that both 31.25 and 15.63 μg/ml remained effective up to 24 h, while 7.81 μg/ml lost activity after 6 h of incubation.

##### 4.1.1.3 Lactobacillus acidophilus TK assay

The TK assay results for NER against *Lactobacillus acidophilus* are presented in Supplementary Table 3. Across all concentrations, bacterial counts varied significantly over time (*P* < 0.001). At 24 h, concentrations ≤ 500 μg/ml showed bacterial counts similar to the growth control (GC). At 2 h=, the 4,000 μg/ml concentration showed higher bacterial counts than 500 and 125 μg/ml (*P* < 0.001). No significant differences were observed among concentrations at 4 h. At 6 h, concentrations between 62.5 and 15.63 μg/ml exhibited greater antimicrobial activity than those ≥2,000 μg/ml (*P* < 0.001). At 24 h, 4,000 μg/ml demonstrated the greatest antimicrobial effect (*P* < 0.001).

Figure 1c shows the TK curves for free NER concentrations encapsulated in the nanoliposome formulation against *L. acidophilus*. All tested concentrations exhibited antimicrobial activity up to 6 h, but this effect was not sustained at 24 h.

#### 4.1.2 Unloaded nanoliposome (UN) and loaded nanoliposome (LN)

##### 4.1.2.1 MRSA TK assay

The TK assay results for all tested concentrations of LN and UN formulations against MRSA are presented in Supplementary Table 4. A significant increase in bacterial counts was observed over time for all concentrations (*P* < 0.001), and none of the formulations maintained antimicrobial activity at 24 h. At 2 and 4 h, the 2,500 μg/ml concentration of LN showed significantly lower bacterial counts compared to all UN concentrations. At 6 h, this concentration also exhibited the highest antimicrobial activity among all tested concentrations of both formulations (*P* < 0.001).

As illustrated in Figure 2, only the 2,500 μg/ml concentration of both LN and UN showed antimicrobial activity up to 6 h. Additionally, while the 1,250 μg/ml and 625 μg/ml LN concentrations remained effective up to 4 h, only the 1,250 μg/ml UN concentration demonstrated similar activity at this time point.

##### 4.1.2.2 E. faecium TK assay

The TK assay results for LN and UN formulations against *Enterococcus faecium* are reported in Supplementary Table 5. All tested concentrations became ineffective after 4 h, with a significant increase in bacterial counts observed over time (*P* < 0.001). No significant differences were found between concentrations at 2 h; however, at 4 h, the 2,500 μg/ml UN concentration exhibited the highest antimicrobial activity (*P* < 0.001).

Figure 3 presents the TK curves for the highest concentrations of the nanoliposome formulations. None of the tested concentrations remained effective beyond 4 h. Notably, for the LN formulation, only the 2,500 μg/ml concentration was active up to 4 h, while all evaluated UN concentrations maintained comparable activity for the same period.

##### 4.1.2.3 L. acidophilus TK assay

The results of the TK assay against *L. acidophilus* are provided in Supplementary Table 6. A significant increase in bacterial counts was observed across all experimental time points (*P* < 0.001). At 2 h, the concentrations of 2,500, 625, and 312.5 μg/ml LN, as well as 156.25 μg/ml LN/UN and all concentrations ≤ 39.09 μg/ml UN, exhibited higher antimicrobial activity compared to 9.77 μg/ml LN (*P* < 0.001). At 4 h, the 2,500, 625, and 312.5 μg/ml UN, along with 625 μg/ml LN, showed significantly lower bacterial counts than 312.5 μg/ml LN, 19.53 μg/ml UN, and 9.77 μg/ml LN (*P* < 0.001). At 6 h, the 2,500 μg/ml LN concentration exhibited greater antimicrobial activity than 156 μg/ml UN and both 39.09 μg/ml LN/UN and concentrations ≤ 19.53 μg/ml LN (*P* < 0.001).

As shown in Figure 4, the highest concentrations of both formulations demonstrated antimicrobial activity up to 6 h; however, none remained effective at 24 h.

##### 4.1.2.4 Salmonella typhimurium TK assay

According to Supplementary Table 7, a significant increase in bacterial counts was observed across the experimental time points for all concentrations ≤ 1,250 μg/ml of both LN and UN formulations (*P* < 0.001). At 2 h, both formulations at 2,500 μg/ml exhibited the strongest antimicrobial activity (*P* < 0.001). This concentration remained effective at 4, 6, and 24 h, with no significant difference in activity observed between LN and UN formulations.

As illustrated in Figure 5, only the 2,500 μg/ml concentration of both LN and UN retained antimicrobial activity throughout the experiment. All concentrations ≤ 1,250 μg/ml were ineffective beyond 2 h.

##### 4.1.2.5 E. coli TK assay

The TK assay data for the highest tested concentrations of LN and UN formulations against *E. coli* are reported in Supplementary Table 8. At 2, 4, and 6 h, the 2,500 μg/ml concentrations of both LN and UN exhibited the highest antimicrobial activity (*P* < 0.001).

Figure 6 displays the TK curves for these concentrations. Only the 2,500 μg/ml LN and UN formulations demonstrated antimicrobial activity up to 6 h; all lower concentrations were ineffective.

### 4.2 Cytotoxicity assay

#### 4.2.1 MTT cell survival assay

**Figure 7:**
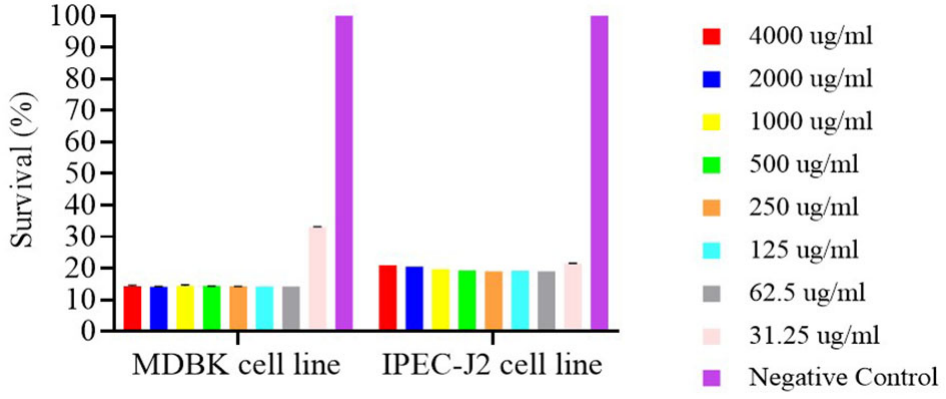
Survival of MDBK and IPEC-J2 cell lines after 24h contact with nerolidol.

The cytotoxic effects of free nerolidol (NER) on MDBK and IPEC-J2 cell lines are presented in Figure 7. Compared to the negative control, all tested concentrations of NER induced significant cytotoxicity in both cell lines (*P* < 0.001). Notably, only in the MDBK cell line was a cell survival rate ≥30% observed at the concentration of 31.25 μg/ml.

**Figure 8:**
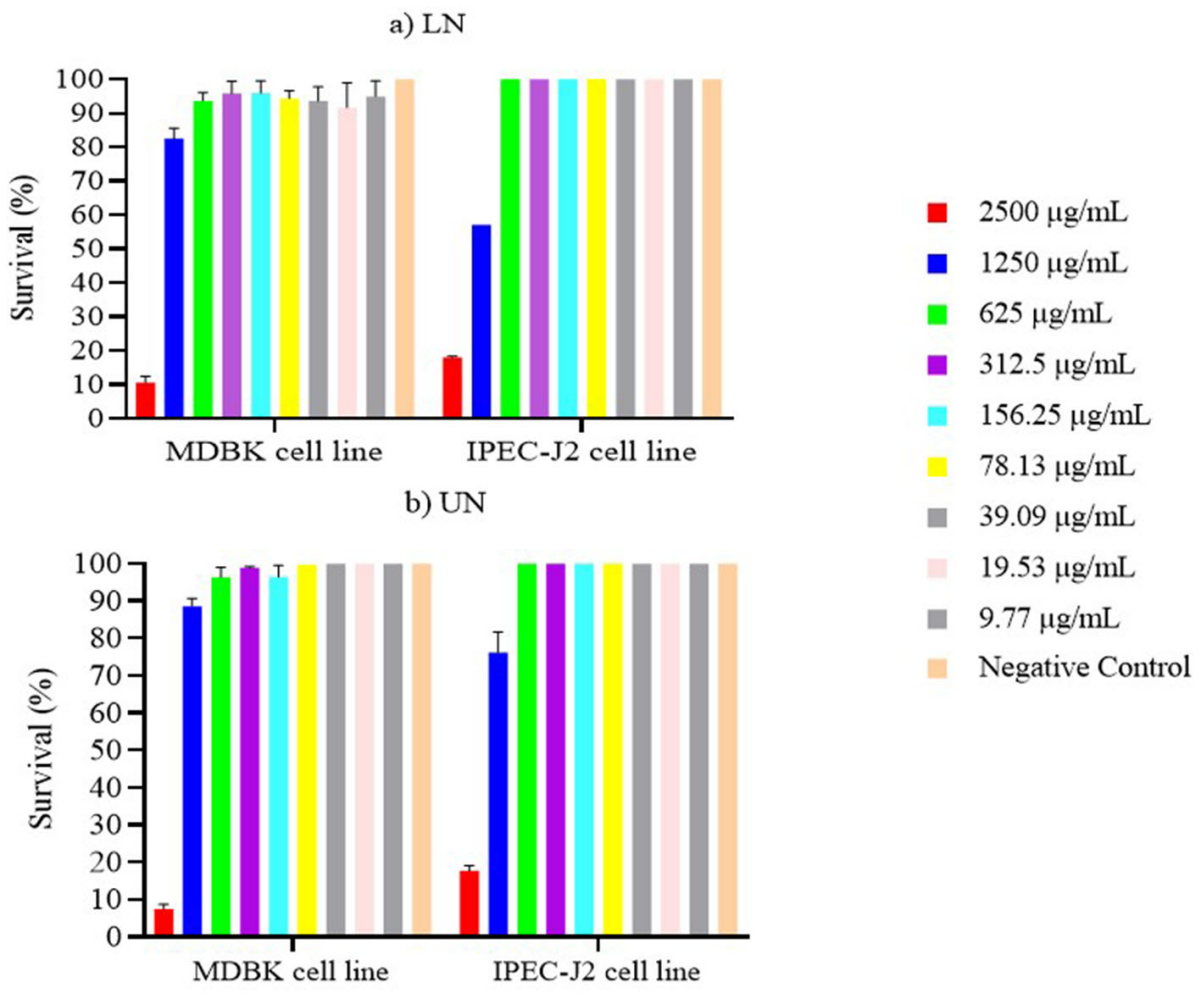
**(a, b)** Survival of MDBK and IPEC-J2 cell lines after 24 h contact with loaded nanoliposome (LN) and unloaded nanoliposome (UN).

The results of the cytotoxicity assays for unloaded nanoliposomes (UN) and nerolidol-loaded nanoliposomes (LN) are shown in Figures 8a, b. Both formulations demonstrated high cytotoxicity at the concentration of 2,500 μg/ml, with survival rates ≤ 10% in both cell lines. In contrast, when cells were exposed to UN and LN at concentrations ≤ 1,250 μg/ml, the MDBK cell line exhibited survival rates above 80%. Furthermore, survival exceeded 90% at concentrations ≤ 625 μg/mL.

Similar results were observed for the IPEC-J2 cell line. Specifically, at concentrations ≤ 625 μg/ml, survival rates approached 100%, with no statistically significant differences between either nanoliposome formulation and the negative control (*P* > 0.05).

## 5 Discussion

PFAs are increasingly studied as alternatives to antibiotics in animal nutrition. However, the potential degradation or structural modifications of NICs and essential oils within the gastrointestinal tract may limit their effectiveness. Encapsulation technologies have been proposed to overcome these limitations, although their efficacy remains a topic of ongoing debate (28–30). In this context, the present study aimed to evaluate a novel feed additive based on nanoliposome loaded with nerolidol, highlighting if the encapsulation enhances the antimicrobial activity of this NIC. For this reason, as a preliminary step, we assessed the antimicrobial activity of free NER across a concentration range of 4,000 to 7.81 μg/ml. Free NER was effective exclusively against Gram-positive bacteria. Interestingly, the highest tested concentration (4,000 μg/ml) showed reduced activity during the initial contact period compared to lower concentrations. This may be attributed to solubility limitations at higher concentrations particularly during the first 6 h of exposure. Additionally, this observation could reflect the so-called ‘Eagle effect'—a paradoxical phenomenon in which higher antimicrobial concentrations result in decreased efficacy, especially when evaluated using time-kill assays (31, 32).

Among the tested bacterial species, the strongest antimicrobial effect after 24 h was observed against *Enterococcus faecium*, with efficacy maintained down to 15.63 μg/ml. Against MRSA, activity was observed up to 62.5 μg/ml. However, NER also demonstrated antimicrobial activity against *Lactobacillus acidophilus*, a beneficial commensal species in the gastrointestinal microbiota of livestock. In this case, a significant reduction in bacterial count was observed at all time points up to 1,000 μg/ml, with the greatest activity at 4,000 μg/ml. By comparing antimicrobial performance across these species, the concentration of 62.5 μg/ml emerged as the lowest dose effective against both pathogenic strains (MRSA and *E. faecium*) while becoming ineffective against *L. acidophilus* after 6 h. These findings underscore the potent, time-dependent antimicrobial activity of NER against Gram-positive bacteria—both pathogenic and commensal—though with varying susceptibility depending on concentration and exposure time. These findings are consistent with previous studies demonstrating the antimicrobial efficacy of free NER, assessed via microdilution assays, against *Staphylococcus aureus* (both methicillin-sensitive and -resistant strains) and *Streptococcus mutans* (13, 14). Conflicting data exists regarding NER activity against *Salmonella enterica*. Some studies report significant antimicrobial effects, while others—aligned with our findings—indicate no activity against Gram-negative species. These discrepancies may be attributed to variations in the botanical source of the NER used (33–35). A proposed mechanism for NER's selective efficacy against Gram-positive bacteria involves its ability to penetrate the bacterial cell wall and disrupt potassium ion homeostasis (13).

Despite the promising antimicrobial profile of NER, its cytotoxicity must be considered as a limiting factor. High cytotoxicity of free NER has been documented in human cell lines, such as HepG2 hepatocellular carcinoma cells, and in model organisms like *Saccharomyces cerevisiae* (13). In our study, NER exhibited pronounced cytotoxicity in both MDBK and IPEC-J2 cell lines, with cell viability dropping below 40% across all tested concentrations. This effect may be explained by the hydrophobic nature of NER, which facilitates its diffusion across cellular membranes and subsequent interaction with intracellular organelles and proteins, ultimately disrupting cellular function (13, 36). These findings emphasize that, despite its antimicrobial potential, the use of free NER in veterinary applications—such as a feed additive—may be limited by its cytotoxic effects.

To enhance the biological properties and reduce the cytotoxicity of NER, the second part of this study focused on incorporating the compound into a nanoliposome system. However, the intrinsic physicochemical characteristics of NER constrained its encapsulation efficiency. As reported in the literature, terpene-loaded liposomes—often referred to as invasomes—typically exhibit low encapsulation capacity, with maximum loading ratios around 1% (36–38). Accordingly, in this study, NER was incorporated at 1% of the total nanoliposome mass (5,000 μg/ml), and tested nanoliposome concentrations were ranging from 2,500 to 9.77 μg/ml, resulting in NER concentrations ranging from 25 to 0.098 μg/ml within the LN (loaded nanoliposome) formulations. Equivalent concentrations were used for the UN (unloaded nanoliposome) formulations as controls. Both LN and UN formulations demonstrated dose- and time-dependent antimicrobial activity against all tested bacteria (Supplementary Tables 4–8). Against MRSA, both formulations showed partial activity for up to 6 h, with LN demonstrating higher efficacy—particularly at 2,500 μg/ml, than UN. Conversely, UN formulations exhibited stronger activity against *Enterococcus faecium*, particularly at the highest concentration and up to 4 h. At lower concentrations, both LN and UN were ineffective. As seen with free-form NER, both nanoliposome formulations also showed antimicrobial activity against *Lactobacillus acidophilus* at early time points (2–6 h), but not at 24 h (Figure 4), suggesting that even low concentrations can impact commensal bacteria in the short term. In the case of Gram-negative bacteria, only the highest concentration (2,500 μg/ml) of both LN and UN was effective. It showed activity against *S*. Typhimurium up to 24 h and *E. coli* up to 6 h, with no notable differences between the two formulations. These findings suggest that both formulations possess non-specific antimicrobial activity. While LN was more effective against MRSA, UN was more potent against *E. faecium* and neither formulation showed greater efficacy than the other against Gram-negative bacteria. These differences may reflect species-specific interactions with nanoliposome structures.

The observed activity of LN and UN against *L. acidophilus* even at the lowest concentrations suggests a lack of selectivity. This aligns with reported non-specific mechanisms of nanoliposome antimicrobial action, including disruption of bacterial membranes, interference with ion regulation, nutrient transport, and induction of oxidative stress. As lipid-based vesicles, nanoliposomes can interact with bacterial membranes—both pathogenic and commensal—through non-specific mechanisms, disrupting the lipid bilayer structure, leading to membrane destabilization and ultimately cell death (39, 40). This disruption can impair vital membrane functions, including nutrient transport, respiration, and ion regulation. Furthermore, liposomes can induce oxidative stress, interfere with bacterial signaling, and enhance immune cell activity (41, 42). These non-specific mechanisms could represent a limitation of these formulations. However, *L. acidophilus* is not the only indicator of healthy microbiota. Other bacteria, such as those belonging to *Bifidobacterium, Firmicutes* and *Bacteroidetes* genera, are also critical for intestinal health but were not tested in the present study. The gut microbiota is in fact a complex and interconnected ecosystem and targeting a single bacterium does not necessarily capture the overall effects on the balance and health of the intestinal environment (43). The impact of PFA on microbiome of animals has been widely studied in literature. For examples as extensively reviewed by Sivamaruthi et al. (44), dietary supplementation with NIC in swine increases appetite improves production performance through positive modulation of the microbiota. Specifically, supplementation with cinnamaldehyde and carvacrol has been shown to increase the abundance of *Lactobacillus* spp., *Prevotella* spp., *Megamonas* spp., *Megasphaera* spp., and *Blautia* spp., while the use of oregano oil decreases the abundance of *Escherichia* and *Shigella* species, which are closely associated with gut dysbiosis. However, there is a lack of information regarding the effects of nerolidol on the livestock microbiome, although the potential impact of nanoliposome formulation has been reported in literature. While traditional antimicrobials disrupt gut microbiota balance and promote opportunistic infections and resistance, nanoliposome formulations present a lower risk of inducing dysbiosis compared to conventional antibiotics (45, 46). This could be partly due to the brief duration of action of these formulations (up to 6 h), which reduces their impact on the gut microbiota, as observed with *L. acidophilus*. Taking all these considerations into account, further studies involving the observation of a wider range of commensal flora and *in vivo* experiments evaluating the impact of nerolidol formulations on gut microbiomes, especially in the long term, are needed to assess the efficacy, tolerability and effects of these formulations on intestinal health status, especially for their non-selective mechanism of action.

To compare free-form NER with the nanoliposome-loaded formulation, only the lower concentrations (≤50 μg/ml) were considered, as these are directly comparable to the LN formulation. The results (Figures 1–6) revealed that LN exhibited a broader antimicrobial spectrum than free-form NER, including activity against Gram-negative bacteria. This could be attributed to the nanoliposome delivery system enhancing NER bioavailability and efficacy. Concerning Gram-positive, encapsulating NER in nanoliposome enhanced its activity against MRSA, reduced its effectiveness against *E. faecium* and showed similar effects on *L. acidophilus*, compared to the free compounds. These results are consistent with previous literature where several studies have compared the antimicrobial effects of encapsulated EOs, plant extracts, or phenolic compounds with their free forms. Some authors report that encapsulating low doses of EOs enhances their antimicrobial activity compared to high doses in free form (28). In contrast, other studies indicate that encapsulating phenolic compounds may reduce their antimicrobial activity due to their slow release from the nanoliposome (29). Therefore, further investigation is needed to better understand the release profile of NER from nanoliposomes and its interaction with microbial targets.

Although soy lecithin—the primary phospholipid in the liposomes—is generally recognized as safe (GRAS) by the FDA, alterations in its chemical composition or the addition of active compounds like NER may influence cytotoxicity. Previous studies indicate that while human cell lines generally tolerate liposomes well, certain cell types, such as L1210 mouse leukemia cells, may be more sensitive to lipid-based vesicles (47). In our study, both LN and UN formulations exhibited dose-dependent cytotoxicity on both MDBK and IPEC-J2 cell lines. Cytotoxicity was slightly higher in LN formulations, likely due to the inclusion of NER. Nonetheless, survival rates remained ≥80% in MDBK cells at concentrations ≤ 1,250 μg/ml, a threshold generally considered acceptable for *in vitro* models (48). Conversely, IPEC-J2 cells were more sensitive to the cytotoxic effects of both nanoliposome formulations at 1,250 μg/ml, with the highest survival rates (100%) observed at concentrations ≤ 625 μg/ml. The slightly higher cytotoxicity of LN formulations compared to UN formulations is due to the presence of NER, which has been shown to exhibit high cytotoxicity, as above mentioned. However, the survival rates in MDBK and IPEC cells at relatively high concentrations suggest good tolerability, which is promising for potential *in vivo* applications.

It is important to consider that determining the appropriate dosage of PFAs remains challenging, as there is often a discrepancy between concentrations shown to be effective (*in vitro* studies) and those that can be safely and practically used *in vivo*. Currently, the dosage of PFAs is not yet fully regulated under Regulation (EC) 1831/2003, since there is no specific category dedicated to them; they are often classified under broader groups such as flavoring or zootechnical additives. As a result, recommended dosages usually rely on manufacturers' guidelines or previous studies (7). For example, doses ranging from 0.5 to 2 g/day of a mixture of thymol, eugenol, vanillin, and limonene have been reported to improve milk production in dairy cows (49). Regarding nerolidol, to the best of our knowledge, no studies have focused on its use in livestock nutrition, though it has been investigated in aquaculture. In a study by Baldisserra et al. (50), nerolidol nanospheres (3 mg/ml) improved body weight in fish fed with 1.0 ml of nanoencapsulated nerolidol per kg of feed compared to a control group. However, significant anatomical and physiological differences exist between mammals (especially ruminants) and fish. Additionally, considering the low oral bioavailability observed in rats due to hepatic metabolism (13), it is crucial to determine the appropriate dosage in cattle, where the rumen could further reduce bioavailability. Therefore, further studies are needed to evaluate the oral administration of nerolidol encapsulated in nanoliposomes for its application in animal nutrition.

## 6 Conclusion

In this study, the antimicrobial effects of NER in its free form and encapsulated in nanoliposomes were evaluated. The results demonstrated that NER, in its free form, exhibits high antimicrobial activity against Gram-positive bacteria, but no efficacy against Gram-negative bacteria. However, its high cytotoxicity limits its applicability, particularly in livestock.

Encapsulation of NER in nanoliposomes enhanced its antimicrobial activity, extending its spectrum to some Gram-negative bacteria, but also revealed activity against the commensal flora, such as *Lactobacillus acidophilus*, suggesting that the formulation is not selective. Both nanoliposome formulations, whether loaded or unloaded with NER, exhibited dose-dependent cytotoxicity, with greater tolerability observed in MDBK cell lines at higher concentrations, suggesting that the use of nanoliposomes may reduce the negative impact on tissues.

The non-specific effects on commensal bacteria highlighted in this study indicate the need for a thorough evaluation of the potential impacts of these formulations on the animal microbiome and their long-term safety. Further studies, particularly *in vivo*, are necessary to assess the safety and efficacy of these formulations, as well as their potential impact on the productive performance and efficiency of livestock when administered diets include these additives.

## Data Availability

The original contributions presented in the study are included in the article/Supplementary material, further inquiries can be directed to the corresponding authors.
