# Supplementary material for: Evaluation of the antimicrobial and cytotoxic activity of nerolidol encapsulated in a nanoliposome system
Source: Front Vet Sci. 2025 Oct 27;12:1641746. doi: 10.3389/fvets.2025.1641746 (PMC12597719; doi:10.3389/fvets.2025.1641746)
Supplement: Supplementary file 1 [file Table_1.docx]

Supplementary Material

**S-Table 1*.*** Time-Kill assay of nerolidol in its free form, against Methicillin-Resistant *Staphylococcus aureus* (MRSA), ATCC 43300.

| µg/mL | Log_10_(CFU/mL) | | | | SEM | P-value |
| --- | --- | --- | --- | --- | --- | --- |
|  | 2h | 4h | 6h | 24h |  |  |
| 4000 | 2.65cCD | 2.78cB | 2.75bB | 0.60aA | 0.215 | ˂0.001 |
| 2000 | 2.58cBC | 2.35cA | 2.16bAB | 0.61aA | 0.171 | ˂0.001 |
| 1000 | 2.48cABC | 2.31cA | 2.01bA | 0.52aA | 0.165 | ˂0.001 |
| 500 | 2.44cABC | 2.29cA | 2.07bA | 0.20aA | 0.190 | ˂0.001 |
| 250 | 2.36bAB | 2.22bA | 2.07bA | 0.48aA | 0.161 | ˂0.001 |
| 125 | 2.34bA | 2.24bA | 2.09bA | 0.48aA | 0.091 | ˂0.001 |
| 62.5 | 2.35bA | 2.23bA | 2.15bAB | 1.34aB | 0.073 | ˂0.001 |
| 31.25 | 2.51aABC | 2.46aAB | 2.50aAB | 4.01bC | 0.151 | ˂0.001 |
| 15.63 | 2.84aD | 3.20bC | 3.48ccC | 4.01dC | 0.089 | ˂0.001 |
| 7.81 | 2.84aD | 3.20bC | 3.48cC | 4.01dC | 0.089 | ˂0.001 |
| SEM | 0.027 | 0.054 | 0.080 | 0.182 |  |  |
| P-value | ˂0.001 | ˂0.001 | ˂0.001 | ˂0.001 |  |  |

Values of 2.84, 3.20, 3.48 and 4.01 Log_10_(CFU/mL) correspond to bacterial growth observed in the growth control. This indicates that at these concentrations, the tested conditions did not exhibit inhibitory effects on bacterial growth*.*

^a–d^ Indicate significant differences within the same concentration at the different experimental time point (P ≤ 0.05).

^A–D^ Indicate significant differences between concentrations at the same experimental time point (P ≤ 0.05).

**S-Table 2*.*** Time-Kill assay of nerolidol in its free form against *Enterococcus faecium* ATCC 19434.

| µg/mL | Log_10_(CFU/mL) | | | | SEM | *P-value* |
| --- | --- | --- | --- | --- | --- | --- |
|  | 2h | 4h | 6h | 24h |  |  |
| 4000 | 2.88bB | 2.88bAB | 2.92bCD | 1.88aC | 0.096 | ˂0.001 |
| 2000 | 2.90bB | 2.96bB | 2.88bBCD | 1.68aBC | 0.115 | ˂0.001 |
| 1000 | 2.79bAB | 2.88bAB | 2.85bABCD | 1.93aCD | 0.083 | ˂0.001 |
| 500 | 2.83bAB | 2.90bAB | 2.81bABC | 1.78aBC | 0.099 | ˂0.001 |
| 250 | 2.78bAB | 2.84bAB | 2.78bABC | 1.71aBC | 0.103 | ˂0.001 |
| 125 | 2.80bAB | 2.74bA | 2.68bAB | 1.71aBC | 0.099 | ˂0.001 |
| 62.5 | 2.68bcA | 2.81cAB | 2.64bA | 1.25aAB | 0.140 | ˂0.001 |
| 31.25 | 2.78bAB | 2.72bA | 2.65bA | 1.06aA | 0.154 | ˂0.001 |
| 15.63 | 2.71abAB | 2.74abA | 2.75bABC | 2.42aD | 0.043 | ˂0.001 |
| 7.81 | 2.72aAB | 2.73aA | 2.99bD | 6.22cE | 0.309 | ˂0.001 |
| SEM | 0.015 | 0.015 | 0.019 | 0.184 |  |  |
| P-value | 0.005 | ˂0.001 | ˂0.001 | ˂0.001 |  |  |

Value of 6.22 Log_10_(CFU/mL) corresponds to bacterial growth observed in the growth control. This indicates that at these concentrations, the tested conditions did not exhibit inhibitory effects on bacterial growth*.*

^a–c^ Indicate significant differences within the same concentration at the different experimental time point (P ≤ 0.05).

^A–E^ Indicate significant differences between concentrations at the same experimental point time (P ≤ 0.05).

**S-Table 3*.*** Time-Kill assay of nerolidol in its free form against *Lactobacillus acidophilus* ATCC 4356.

| µg/mL | Log_10_(CFU/mL) | | | | SEM | P-value |
| --- | --- | --- | --- | --- | --- | --- |
|  | 2h | 4h | 6h | 24h |  |  |
| 4000 | 2.51aC | 2.32a | 2.92cBC | 2.72bA | 0.052 | ˂0.001 |
| 2000 | 2.25aABC | 2.25a | 2.90bBC | 3.00cB | 0.078 | ˂0.001 |
| 1000 | 2.45aBC | 2.30a | 2.84cABC | 3.08dB | 0.067 | ˂0.001 |
| 500 | 2.19aAB | 2.26a | 2.85bABC | 7.14cC | 0.429 | ˂0.001 |
| 250 | 2.27aABC | 2.19a | 2.79bAB | 7.14cC | 0.429 | ˂0.001 |
| 125 | 2.02aA | 2.33b | 2.75cAB | 7.14dC | 0.435 | ˂0.001 |
| 62.5 | 2.26aABC | 2.32a | 2.71bA | 7.14cC | 0.427 | ˂0.001 |
| 31.25 | 2.23aABC | 2.16a | 2.76bA | 7.14cC | 0.432 | ˂0.001 |
| 15.63 | 2.42aBC | 2.18a | 2.72cA | 7.14dC | 0.426 | ˂0.001 |
| 7.81 | 2.29aABC | 2.29a | 2.99bC | 7.14cC | 0.422 | ˂0.001 |
| SEM | 0.025 | 0.02 | 0.015 | 0.251C |  |  |
| P-value | ˂0.001 | 0.544 | ˂0.001 | ˂0.001 |  |  |

Value of 7.14 Log_10_(CFU/mL) corresponds to bacterial growth observed in the growth control. This indicates that at these concentrations, the tested conditions did not exhibit inhibitory effects on bacterial growth*.*

^a–d^ Indicate significant differences within the same concentration at the different experimental time point (P ≤ 0.05).

^A–C^ Indicate significant differences between concentrations at the same experimental point time (P ≤ 0.05).

**S-Table 4*.*** Time-Kill assay of loaded nanoliposome (LN) and unloaded nanoliposome (UN), against Methicillin-Resistant *Staphylococcus aureus* (MRSA) ATCC 43300.

| µg/mL | Log_10_(CFU/mL) | | | SEM | P-value |
| --- | --- | --- | --- | --- | --- |
|  | 2h | 4h | 6h |  |  |
| 2500 LN | 2.58aA | 2.81bA | 3.30cA | 0.074 | ˂0.001 |
| 2500 UN | 2.72aBCD | 2.91bB | 3.42cB | 0.064 | ˂0.001 |
| 1250 LN | 2.64aAB | 2.83bAB | 3.48cB | 0.086 | ˂0.001 |
| 1250 UN | 2.74aBCD | 3.08bC | 3.48cB | 0.073 | ˂0.001 |
| 625 LN | 2.70aABC | 2.98bBC | 3.48cB | 0.080 | ˂0.001 |
| 625 UN | 2.77aC | 3.20bD | 3.48cB | 0.071 | ˂0.001 |
| 312.5 LN | 2.74aBCD | 3.08bD | 3.48cB | 0.074 | ˂0.001 |
| 312.5 UN | 2.79aCD | 3.20bD | 3.48cB | 0.069 | ˂0.001 |
| 156.25 LN | 2.76aBCD | 3.20bD | 3.48cB | 0.073 | ˂0.001 |
| 156.25 UN | 2.81aCD | 3.20bD | 3.48cB | 0.067 | ˂0.001 |
| 78.13 LN | 2.84aD | 3.20bD | 3.48cB | 0.066 | ˂0.001 |
| 78.13 UN | 2.84aD | 3.20bD | 3.48cB | 0.063 | ˂0.001 |
| 39.09 LN | 2.84aD | 3.20bD | 3.48cB | 0.063 | ˂0.001 |
| 39.09 UN | 2.84aD | 3.20bD | 3.48cB | 0.063 | ˂0.001 |
| 19.53 LN | 2.84aD | 3.20bD | 3.48cB | 0.063 | ˂0.001 |
| 19.53 UN | 2.84aD | 3.20bD | 3.48cB | 0.063 | ˂0.001 |
| 9.77 LN | 2.84aD | 3.20bD | 3.48cB | 0.063 | ˂0.001 |
| 9.77 UN | 2.84aD | 3.20bD | 3.48cB | 0.063 | ˂0.001 |
| SEM | 0.009 | 0.013 | 0.005 |  |  |
| P-value | ˂0.001 | ˂0.001 | ˂0.001 |  |  |

Values of 2.84, 3.20, and 3.48 Log_10_(CFU/mL) correspond to bacterial growth observed in the growth control. This indicates that at these concentrations, the tested conditions did not exhibit inhibitory effects on bacterial growth*.*

^a–c^ Indicate significant differences within the same concentration at the different experimental time point (P ≤ 0.05).

^A–D^ Indicate significant differences between concentrations at the same experimental point time (P ≤ 0.05).

**S-Table 5.** Time-Kill assay of loaded nanoliposome (LN) and unloaded nanoliposome (UN), against *Enterococcus faecium* ATCC 19434.

|  |  |  |  |  |
| --- | --- | --- | --- | --- |
| µg/mL | Log_10_(CFU/mL) | | SEM | P-value |
|  | 2h | 4h |  |  |
| 2500 LN | 2.39a | 3.01bC | 0.051 | ˂0.001 |
| 2500 UN | 2.32a | 2.82bA | 0.077 | ˂0.001 |
| 1250 LN | 2.37a | 4.18bE | 0.098 | ˂0.001 |
| 1250 UN | 2.34a | 2.92bB | 0.089 | ˂0.001 |
| 625 LN | 2.42a | 4.18bF | 0.265 | ˂0.001 |
| 625 UN | 2.38a | 3.06bD | 0.103 | ˂0.001 |
| 312.5 LN | 2.40a | 4.18bF | 0.269 | ˂0.001 |
| 312.5 UN | 2.33a | 4.18bF | 0.279 | ˂0.001 |
| 156.25 LN | 2.36a | 4.18bF | 0.275 | ˂0.001 |
| 156.25 UN | 2.38a | 4.18bF | 0.272 | ˂0.001 |
| 78.13 LN | 2.41a | 4.18bF | 0.268 | ˂0.001 |
| 78.13 UN | 2.39a | 4.18bF | 0.269 | ˂0.001 |
| 39.09 LN | 2.48a | 4.18bF | 0.257 | ˂0.001 |
| 39.09 UN | 2.36a | 4.18bF | 0.274 | ˂0.001 |
| 19.53 LN | 2.36a | 4.18bF | 0.269 | ˂0.001 |
| 19.53 UN | 2.37a | 4.18bF | 0.274 | ˂0.001 |
| 9.77 LN | 2.34a | 4.18bF | 0.276 | ˂0.001 |
| 9.77 UN | 2.35a | 4.18bF | 0.275 | ˂0.001 |
| SEM | 0.010 | 0.002 |  |  |
| P-value | 0.667 | ˂0.001 |  |  |

Value of 4.18 Log_10_(CFU/mL) corresponds to bacterial growth observed in the growth control. This indicates that at these concentrations, the tested conditions did not exhibit inhibitory effects on bacterial growth*.*

^a–b^ Indicate significant differences within the same concentration at the different experimental time point (P ≤ 0.05).

^A–F^ Indicate significant differences between concentrations at the same experimental point time (P ≤ 0.05).

S-Table 6. Time-Kill assay of loaded nanoliposome (LN) and unloaded nanoliposome (UN), against *Lactobacillus acidophilus* ATCC 4356.

|  |  |  |  |  |  |
| --- | --- | --- | --- | --- | --- |
| µg/mL | Log_10_(CFU/mL) | | | SEM | P-value |
|  | 2h | 4h | 6h |  |  |
| 2500 LN | 2.04aA | 2.30bABC | 2.65cA | 0.062 | ˂0.001 |
| 2500 UN | 2.27aAB | 2.25aA | 2.69bAB | 0.060 | ˂0.001 |
| 1250 LN | 2.23aAB | 2.28aAB | 2.77bABC | 0.060 | ˂0.001 |
| 1250 UN | 2.34aAB | 2.34aABC | 2.79bABC | 0.074 | ˂0.001 |
| 625 LN | 1.98aA | 2.17aA | 2.81bABC | 0.065 | ˂0.001 |
| 625 UN | 2.27aAB | 2.26aA | 2.78bABC | 0.093 | ˂0.001 |
| 312.5 LN | 2.12aA | 2.55bBC | 2.85cABC | 0.069 | ˂0.001 |
| 312.5 UN | 2.21aAB | 2.20aA | 2.76bABC | 0.076 | ˂0.001 |
| 156.25 LN | 2.02aA | 2.31bABC | 2.82cABC | 0.082 | ˂0.001 |
| 156.25 UN | 2.10aA | 2.49bABC | 2.91cBC | 0.087 | ˂0.001 |
| 78.13 LN | 2.26aAB | 2.28aAB | 2.88bABC | 0.072 | ˂0.001 |
| 78.13 UN | 2.25aAB | 2.63bC | 2.78bABC | 0.056 | ˂0.001 |
| 39.09 LN | 2.19aAB | 2.48bABC | 2.97cC | 0.079 | ˂0.001 |
| 39.09 UN | 2.12aA | 2.50bABC | 2.96cC | 0.089 | ˂0.001 |
| 19.53 LN | 2.22aAB | 2.40aABC | 2.96bC | 0.082 | ˂0.001 |
| 19.53 UN | 2.08aA | 2.57bBC | 2.89cABC | 0.086 | ˂0.001 |
| 9.77 LN | 2.39aB | 2.61bBC | 2.94cC | 0.067 | ˂0.001 |
| 9.77 UN | 2.06aA | 2.40bA | 2.90cABC | 0.097 | ˂0.001 |
| SEM | 0.016 | 0.019 | 0.013 |  |  |
| P-value | ˂0.001 | ˂0.001 | ˂0.001 |  |  |

^a–c^ Indicate significant differences within the same concentration at the different experimental time point (P ≤ 0.05).

^A–C^ Indicate significant differences between concentrations at the same experimental point time (P ≤ 0.05).

S-Table 7. Time-Kill assay of loaded nanoliposome (LN) and unloaded nanoliposome (UN), against *Salmonella* Typhimurium ATCC 14028.

| µg/mL | Log_10_(CFU/mL) | | | | SEM | P-value |
| --- | --- | --- | --- | --- | --- | --- |
|  | 2h | 4h | 6h | 24h |  |  |
| 2500 LN | 2.97bA | 3.10dA | 3.07cA | 2.58aA | 0.045 | ˂0.001 |
| 2500 UN | 2.87aA | 3.09cA | 3.09cA | 2.48aA | 0.053 | ˂0.001 |
| 1250 LN | 3.11aB | 5.69bB | 6.20cB | 7.14dB | 0.357 | ˂0.001 |
| 1250 UN | 3.29aB | 5.69bB | 6.20cB | 7.14dB | 0.351 | ˂0.001 |
| 625 LN | 3.77bC | 5.69bB | 6.20cB | 7.14dB | 0.312 | ˂0.001 |
| 625 UN | 4.32aD | 5.69bB | 6.20cB | 7.14dB | 0.323 | ˂0.001 |
| 312.5 LN | 4.32aD | 5.69bB | 6.20cB | 7.14dB | 0.263 | ˂0.001 |
| 312.5 UN | 4.32aD | 5.69bB | 6.20cB | 7.14dB | 0.211 | ˂0.001 |
| 156.25 LN | 4.32aD | 5.69bB | 6.20cB | 7.14dB | 0.205 | ˂0.001 |
| 156.25 UN | 4.32aD | 5.69bB | 6.20cB | 7.14dB | 0.211 | ˂0.001 |
| 78.13 LN | 4.32aD | 5.69bB | 6.20cB | 7.14dB | 0.211 | ˂0.001 |
| 78.13 UN | 4.32aD | 5.69bB | 6.20cB | 7.14dB | 0.211 | ˂0.001 |
| 39.09 LN | 4.32aD | 5.69bB | 6.20cB | 7.14dB | 0.211 | ˂0.001 |
| 39.09 UN | 4.32aD | 5.69bB | 6.20cB | 7.14dB | 0.211 | ˂0.001 |
| 19.53 LN | 4.32aD | 5.69bB | 6.20cB | 7.14dB | 0.211 | ˂0.001 |
| 19.53 UN | 4.32aD | 5.69bB | 6.20cB | 7.14dB | 0.211 | ˂0.001 |
| 9.77 LN | 4.32aD | 5.69bB | 6.20cB | 7.14dB | 0.211 | ˂0.001 |
| 9.77 UN | 4.32aD | 5.69bB | 6.20cB | 7.14dB | 0.211 | ˂0.001 |
| SEM | 0.064 | 0.103 | 0.119 | 0.139 |  |  |
| P-value | ˂0.001 | ˂0.001 | ˂0.001 | ˂0.001 |  |  |

Values of 4.33, 5.69, 6.20 and 7.14 Log_10_(CFU/mL) corresponds to bacterial growth observed in the growth control. This indicates that at these concentrations, the tested conditions did not exhibit inhibitory effects on bacterial growth*.*

^a–d^ Indicate significant differences within the same concentration at the different experimental time point (P ≤ 0.05).

^A–D^ Indicate significant differences between concentrations at the same experimental point time (P ≤ 0.05).

S-Table 8. Time-Kill assay of loaded nanoliposome (LN) and unloaded nanoliposome (UN), against *Escherichia coli* ATCC 25922.

| µg/mL | Log_10_(CFU/mL) | | | SEM | P-value |
| --- | --- | --- | --- | --- | --- |
|  | 2h | 4h | 6h |  |  |
| 2500 LN | 2.74aA | 3.09cA | 3.00bA | 0.044 | ˂0.001 |
| 2500 UN | 2.69aA | 3.08cA | 3.00bA | 0.051 | ˂0.001 |
| 1250 LN | 3.03aB | 5.70bB | 6.04cB | 0.327 | ˂0.001 |
| 1250 UN | 3.11aB | 5.70bB | 6.04cB | 0.317 | ˂0.001 |
| 625 LN | 3.11aB | 5.70bB | 6.04cB | 0.318 | ˂0.001 |
| 625 UN | 3.11aB | 5.70bB | 6.04cB | 0.317 | ˂0.001 |
| 312.5 LN | 3.11aB | 5.70bB | 6.04cB | 0.317 | ˂0.001 |
| 312.5 UN | 3.11aB | 5.70bB | 6.04cB | 0.317 | ˂0.001 |
| 156.25 LN | 3.11aB | 5.70bB | 6.04cB | 0.317 | ˂0.001 |
| 156.25 UN | 3.11aB | 5.70bB | 6.04cB | 0.317 | ˂0.001 |
| 78.13 LN | 3.11aB | 5.70bB | 6.04cB | 0.317 | ˂0.001 |
| 78.13 UN | 3.11aB | 5.70bB | 6.04cB | 0.317 | ˂0.001 |
| 39.09 LN | 3.11aB | 5.70bB | 6.04cB | 0.317 | ˂0.001 |
| 39.09 UN | 3.11aB | 5.70bB | 6.04cB | 0.317 | ˂0.001 |
| 19.53 LN | 3.11aB | 5.70bB | 6.04cB | 0.317 | ˂0.001 |
| 19.53 UN | 3.11aB | 5.70bB | 6.04cB | 0.317 | ˂0.001 |
| 9.77 LN | 3.11aB | 5.70bB | 6.04cB | 0.317 | ˂0.001 |
| 9.77 UN | 3.11aB | 5.70bB | 6.04cB | 0.317 | ˂0.001 |
| SEM | 0.013 | 0.080 | 0.092 |  |  |
| P-value | ˂0.001 | ˂0.001 | ˂0.001 | |  |

Values of 3.11, 5.70 and 6.04 Log_10_(CFU/mL) corresponds to bacterial growth observed in the growth control. This indicates that at these concentrations, the tested conditions did not exhibit inhibitory effects on bacterial growth*.*

^a–c^ Indicate significant differences within the same concentration at the different experimental time point (P ≤ 0.05).

^A–B^ Indicate significant differences between concentrations at the same experimental point time (P ≤ 0.05).
